# Intramammary infusion of matrine-chitosan hydrogels for treating subclinical bovine mastitis —effects on milk microbiome and metabolites

**DOI:** 10.3389/fmicb.2022.950231

**Published:** 2022-09-20

**Authors:** Hua Zhang, Ziyue Wang, Hua Yao, Linshu Jiang, Jinjin Tong

**Affiliations:** Animal Science and Technology College, Beijing University of Agriculture, Beijing, China

**Keywords:** matrine-chitosan hydrogels, inflammatory factors, mammary gland, immunity, microbiome, metabolomics

## Abstract

**Background:**

Bovine metabolism undergoes significant changes during subclinical mastitis, but the relevant molecular mechanisms have not been elucidated. In this study we investigated the changes in milk microbiota and metabolites after intramammary infusion of matrine-chitosan hydrogels (MCHs) in cows with subclinical mastitis.

**Methods:**

Infusions were continued for 7 days, and milk samples were collected on days 1 and 7 for microbiome analysis by 16S rRNA gene sequencing and metabolite profiling by liquid chromatography-mass spectrometry.

**Results:**

MCHs significantly decreased the somatic cell count on day 7 compared to day 1, and the Simpson index indicated that microbial diversity was significantly lower on day 7. The relative abundance of *Aerococcus*, *Corynebacterium*_1, *Staphylococcus* and *Firmicutes* was significantly decreased on day 7, while Proteobacteria increased. In the milk samples, we identified 74 differentially expressed metabolites. The MCHs infusion group had the most significantly upregulated metabolites including sphingolipids, glycerophospholipids, flavonoids and fatty acyls. The mammary gland metabolic pathways identified after MCHs treatment were consistent with the known antimicrobial and anti-inflammatory properties of matrine that are associated with glycerophospholipid metabolism and the sphingolipid metabolic signaling pathways.

**Conclusion:**

These insights into the immunoregulatory mechanisms and the corresponding biological responses to matrine demonstrate its potential activity in mitigating the harmful effects of bovine mastitis.

## Introduction

The udder microbiota plays an important role in the host-pathogen interactions of the innate and adaptive immune system. This is especially true of pathogens that trigger inflammation that is detrimental to mammary tissue and bovine physiology (Gao et al., 2017; Derakhshani et al., 2018). Mastitis is an inflammation of the udder most commonly from bacterial infection, and the pathological changes not only affect milk quality but also quantity (Gao et al., 2017). The increasing demand for animal protein has led to a substantial increase in the use of antibiotics in food animal production (Guillaume et al., 2016). This can exacerbate antimicrobial resistance, which is a global concern for treating human as well as farm animal diseases. Developing an effective drug for mastitis that is safe for use in food animals and for which no resistance exists in the microbial population would be highly desirable for the worldwide dairy industry.

Matrine is a polycyclic alkaloid isolated from the plant, *Sophora flavescens*, that has been used in Chinese traditional medicine because of its anticancer, anti-inflammatory, cardioprotective, and opioid effects (Xiaofei et al., 2018). Previous research (Feng et al., 2018) found that matrine inhibited the virulence of *Staphylococcus aureus*, one of the main causes of mastitis. Matrine attenuated the lipopolysaccharide-induced immune response by downregulating IL-1 and IL-17 and inhibiting production of the proinflammatory compound, malondialdehyde, reducing inflammation and oxidative stress, and enhancing CCR7 expression (Zhou et al., 2017; Sun et al., 2018a). Chitosan is a natural polymer produced by acetylation and enzymatic cleavage of chitin, the most abundant animal polysaccharide in nature (Zhang et al., 2019). The applications for chitosan have continued to increase because of its biocompatibility and ease of chemical modification, together with its anti-inflammatory, antimicrobial, cholesterol-lowering, immunomodulatory, and antitumor properties (Feng et al., 2018). Chitosan can be beneficial at the cellular or molecular level (Sun et al., 2018b), by reducing intracellular material leakage and triggering an antimicrobial response to enhance the therapeutic effects of mastitis treatment. Chitosan can be formulated to be injectable at room temperature, but change into a biodegradable hydrogel at body temperature (Chenite et al., 2000). It has been used in drug formulations and delivery vehicles for decades; However, the influence of matrine and chitosan on the udders of dairy cows has received little research attention. Therefore, it is worthwhile to explore the effectiveness of intramammary infusion of matrine-loaded chitosan hydrogels (MCHs) on the udders of dairy cows with mastitis in terms of positively changing the microbial population and metabolic profile of milk. We hypothesized that intramammary infusion of MCHs would favor the growth of beneficial microorganisms and cause a shift in diversity of the milk microbiota in normal udder quarters.

DNA sequencing has become routine for many labs and the advanced software and bioinformatics databases available have created great opportunities for studying pathogenic mechanisms (Oikonomou et al., 2014; Falentin et al., 2016). By understanding the host’s responses to microbial attack, we expected to be able to identify the most effective targets for antimicrobial intervention, to develop new treatments for bovine mastitis and to find sensitive biomarkers for early detection and diagnosis. Metabolomics can also be used for quantitative measurement of the metabolic state of milk, including the biomarkers of lactation (Sun et al., 2017), the variation in metabolites associated with mastitis (Tong et al., 2019), and the changes in metabolism after antibacterial therapy (Junza et al., 2016). Little is known about the changes in milk metabolites in response to MCHs treatment of mastitis; Therefore, one of the goals of this study was to obtain detailed information on the effect of matrine delivery by chitosan hydrogel infusion into bovine mammary glands on milk microbiota and metabolites. We investigated the effect of MCHs on subclinical-mastitis and the relationship between milk microbiota diversity and metabolite profiles, to probe the mechanisms of the antibacterial benefits of matrine and chitosan for mastitis. The results can be provided new knowledge for the development of novel, safe and effective prophylactic, and therapeutic compounds for dairy cow operations.

## Materials and methods

### Preparation of matrine-loaded chitosan hydrogels

Matrine (98% purity) was purchased from Sigma-Aldrich Corp. (St. Louis, MO, United States), and chitosan was purchased from Shanghai Sunny Biotech Co., Ltd., Shanghai, China. All solutions were prepared with nonpyrogenic products and materials under aseptic conditions in a laminar flow hood. The procedures were like those of Lanct et al. (2017) and Jt et al. (2022). Matrine (0.05 g) and chitosan (4 g) were added to 95 ml of deionized water and heated at 37°C until the chitosan dissolved to form matrine-loaded chitosan complexes. Ten ml of matrine-chitosan and 5 ml of 4% hydroxyethyl cellulose (2:1 ratio) were put into disposable plastic dishes (60 mm diameter) and mixed thoroughly. The water used was nonpyrogenic with <0.005 endotoxin units/mL (Lonza, Walkersville, MD). For intramammary infusion, plastic syringes were filled with 10 ml of MCHs, sealed and stored at room temperature.

### Animals and experimental design

Animals selected in this study were provided by the Beijing Sunlow Livestock Dairy Farming Center (Beijing, China), our animal protocols were reviewed and accepted by the Animal Care Committee of Beijing University of Agriculture (BUA2021056, Beijing, China) in concordance with the guidelines for the use of bovines in research studies of the SSTCC (The State Science and Technology Commission of the P.R. of China, 2017). Samples of untreated milk (*n* = 580) were received from commercial dairies in Beijing from June to August 2021 with the California mastitis rapid detection reagent (CMT). Twelve mid-lactation cows with high somatic cell counts (SCC, average 360,000 cells/ml) indicative of subclinical-mastitis were selected according to milk yield and parity. There were few initial differences in milk yield (24.2 ± 2.7 kg/d), DIM (127 ± 14.4 d), parity (2.3 ± 0.2), or body weight (BW; 726 ± 27 kg). The cows were fed a standard basal diet (Table 1) with free access to water, and were kept in a tie-stall barn. They received intramammary infusions either MCHs or CON (ceftiofur hydrochloride, 125 mg, Zoetis, Florham Park, NJ, United States) after each milking, twice daily (7 AM and 7 PM) for 7 days. Milk samples were collected on the first day (D1) and the last injection day (D7) and totaled 15 ml per animal, with about equal volumes from each lactating udder quarter. The milk samples were cleaned and sterilized by hand after discarding the first three milkings. SCCs were determined using an automatic cell counter (DeLaval International AB, Tumba, Sweden), and the remaining milk was stored in liquid nitrogen for later analysis. At the end of the experimental period, all cows were returned to the herd after veterinary examination ensured they were healthy.

**Table 1:** Components and nutritional analysis of the total mixed rations (dry matter basis, *n* = 6).

**Item**	**Content (%)**
Alfalfa hay	13.34
Leymus chinensis	11.20
Corn	15.73
Whole cottonseed	3.19
Maize silage	28.57
DDGS	2.99
Steam-flaked corn	7.16
Soybean meal	11.53
Cottonseed meal	3.87
Premix^1^	1.96
NaCl	0.46
**Total**	100.00
**Nutrient levels**	
NEL/(MJ/kg)^2^	7.26
EE	4.97
CP	17.35
NDF	30.8
ADF	16.5
Ca	0.74
P	0.41

1 One kg of premix contained the following: Cu 1,230 mg, Zn 4,950 mg, Mn 1760 mg, I 50 mg, Se 61 mg, VA 230000 IU, VD 350000 IU, VE 1000 IU.

2 NEL was a calculated value; the other nutrient levels were measured values.

### Isolation of bacterial DNA and sequencing of 16S rRNA genes

Bacterial DNA was isolated from milk using a Power Soil DNA isolation kit (Qiagen, United Kingdom) as previously described by Tong et al. (2019). DNA concentration and 260/280 ratio were measured with a NanoDrop 1,000 spectrophotometer (Nanodrop Technologies, United States), and integrity was visualized by running DNA aliquots on an agarose gel. The V1-V2 region of the 16S rRNA gene was PCR-amplified with a GeneAmp 9,700 (ABI, United States) using forward (5’-CGTATCGCCT-CCCTCGCGCCATCAG-3′) and reverse (5’-CTA-TGCGCCTTGCCAGCCCGCTCAG-3′) primers that incorporated adaptors and barcodes (Tong et al., 2019). Amplicons of about 450 bp were selected and combined in equal concentrations (Li Y. et al., 2018), then electrophoresed and extracted from the gel with GeneJET (Thermo-Fisher, Waltham, MA, United States). Paired-end libraries were prepared by Majorbio Bio-Pharm (Shanghai, China). Bacterial rRNA genes were sequenced with Illumina HiSeq (Illumina, United States) to obtain paired-end reads of 300 base pairs. All raw sequence data were uploaded to NCBI (#SRP254162).

### Analytical bioinformatics of milk microbiota

Analyses were conducted with FLASH version 1.2.11 and quantitative insights into microbial ecology (QIIME) version 1.9.1. These programs gave data like that published by Tong et al. (2019). Sequences were assigned to taxa by BLASTing the ribosomal database project (RDP) dbase using a 97% identity cut-off. Operational taxonomic units (OTUs) were normalized to relative abundance and bacterial composition was determined by Majorbio I-Sanger. Within-sample diversity (α-diversity) was measured as bacterial community enrichment (ACE and Chao indices) and diversity (Shannon and Simpson indices) was measured in a stochastic subset of the OTUs. Between-sample microbial diversity (β-diversity) was determined by phylogenetically-based, weighted UniFrac distances (Lozupone and Knight, 2005). A more detailed picture of the diversity of the most abundant evolutionary clades in the bovine microbiota was obtained by filtering the OTUs to yield those with a relative abundance of ≥1% for at least one sample.

### Milk metabolome determination

Milk samples were assessed by Majorbio Bio-Pharm using the LC–MS AB Sciex Triple TOF 5600TM (AB SCIEX, United States) according to published procedures (Tong et al., 2019). The liquid chromatography conditions were like those of a previous study (Wang et al., 2019). Quality controls were run by combining milk samples and injecting them periodically during experimental measurements. The results were analyzed with XCMS (ver. 3.4.4). The retention times, MZ, observations, and peak intensities were normalized with Excel. The differentially expressed metabolites were analyzed with the public database^1^ on the Majorbio I-Sanger platform^2^ and the KEGG pathway software for differential metabolite profiles.^3^

### Multivariate statistics

Comparisons were validated by Student’s *t*, and *p* < 0.05 was defined as significant. Hierarchical clustering was conducted using the Bray–Curtis similarity index and the unweighted pair-group method with arithmetic averages. The SPSS software v.21.0 (IBM, Armonk, NY) was used. The α-diversity indices were given as mean ± SD. A *p* < 0.05 was considered statistically significant, and a *p* < 0.10 suggested a trend. Principal component analysis and orthogonal partial least-squares-discriminant analysis (OPLS-DA) were carried out to show the metabolism changes among the experimental groups after mean centering and unit variance scaling. Parameters with variable importance in projection (VIP) values >1.0 were allowed for group discriminant testing. Our OPLS-DA model was confirmed by 7-fold permutation testing. Significantly different metabolites among groups were assessed and identified by Wilcoxon rank-sum tests.

## Results

### Effect of matrine-chitosan hydrogels on milk yield and milk composition

Compared with D1, the milk yield, the yield of milk fat (*p* = 0.03), protein (*p* = 0.04), lactose (*p* = 0.04), and energy-corrected milk (ECM; *p* = 0.01) content all significantly increased by D7 (Table 2). MCHs significantly decreased the SCC on D7 compared to D1 (*p* < 0.01). However, there were no differences among the percentages of lactose, fat, and protein (*p* > 0.05). Similar results were found in CON group.

**Table 2:** Comparisons of milk yield and composition after intramammary treatment with matrine-loaded chitosan hydrogel (*n* = 6).

**Yield and composition**	**MCH**				**CON**			
	**D1**	**D7**	**SEM**	***P*-value**	**D1**	**D7**	**SEM**	***P*-value**
**Yield**								
Milk yield (kg/day)	25.2	34.27	2.67	0.03	23.17	29.93	2.81	0.05
ECM^1^ (kg/day)	27.3	39.2	2.18	0.01	24.10	32.35	3.16	0.05
Lactose (kg/day)	1.17	1.67	0.17	0.04	1.09	1.57	0.17	0.04
Fat (kg/day)	0.97	1.4	0.07	<0.01	0.85	1.14	0.12	0.05
Protein (kg/day)	0.8	1.23	0.14	0.04	0.68	0.96	0.09	0.04
**Composition**								
Lactose (%)	4.59	4.82	0.17	0.25	4.58	5.26	0.29	0.05
Fat (%)	3.87	4.13	0.28	0.41	3.62	3.83	0.17	0.23
Protein (%)	3.12	3.53	0.2	0.08	2.95	3.21	0.09	0.04
Fat: protein	1.17	1.17	0.09	0.99	1.23	1.20	0.07	0.60
SCC (×10^4^ cells/ml)	35.4	10.7	3.6	<0.01	37.59	15.25	2.33	<0.01

### Diversity and relative numbers of milk microbiota

**Figure 1:**
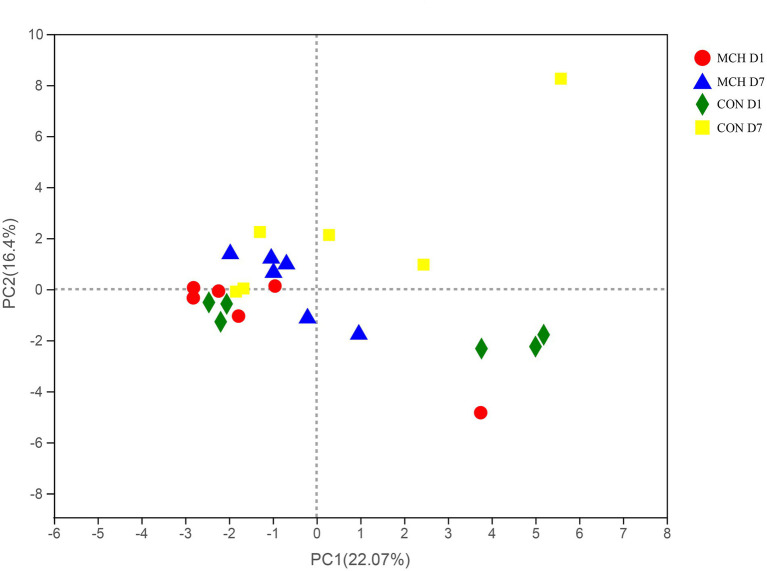
PCA of milk microbial communities from cows with subclinical mastitis between MCH group and CON group from day 1 to day 7 (*n* = 6).

A total of 950,855 high-quality sequences from 24 samples passed quality control and were used for testing. The sequences averaged 460 bp, and there was greater than 99 % depth coverage; Therefore, the amount of data was sufficient to show differences in all bacterial species. The α-diversity indices of the microbiota are shown in Table 3. Although ACE showed a tendency to decrease, no difference was observed in the Chao indices, which were all representative of bacterial community richness in MCH group. Moreover, the Simpson index showed that the bacterial diversity at D7 was significantly higher than at D1 (*P* < 0.05) in both groups, and there was an overall tendency toward decrease (*p* = 0.06), but this was variable and depended on whether the calculations were based on abundance or biomass. No significant differences were seen among the groups for the other α-diversities. The PCA scores showed clear differences between MCH group and CON group on D1 and D7 milk samples (Figure 1). Principal components one and two were found to account for 22.07 and 16.4% of the variation, respectively.

**Table 3:** Alpha diversity indices for milk microbiota.

**Item**	**MCH**			**CON**		
	**D 1**	**D 7**	***P*-value**	**D 1**	**D 7**	***P*-value**
ACE	1769.86 ± 189.07	1211.79 ± 196.29	0.08	1659.6 ± 257.01	1,096 ± 597.53	0.06
Chao	1675.43 ± 201.72	1176.47 ± 196.24	0.11	1439.50 ± 437.05	1025.4 ± 573.23	0.19
Simpson	0.19 ± 0.07	0.47 ± 0.09	0.05^*^	0.15 ± 0.12	0.37 ± 0.24	0.07
Shannon	3.35 ± 0.52	1.92 ± 0.36	0.06	3.53 ± 0.82	2.22 ± 0.90	0.03^*^
Coverage	0.99 ± 0.01	0.99 ± 0.02	0.57	0.99 ± 0.01	0.99 ± 0.01	0.14

*p < 0.05.

**Figure 2:**
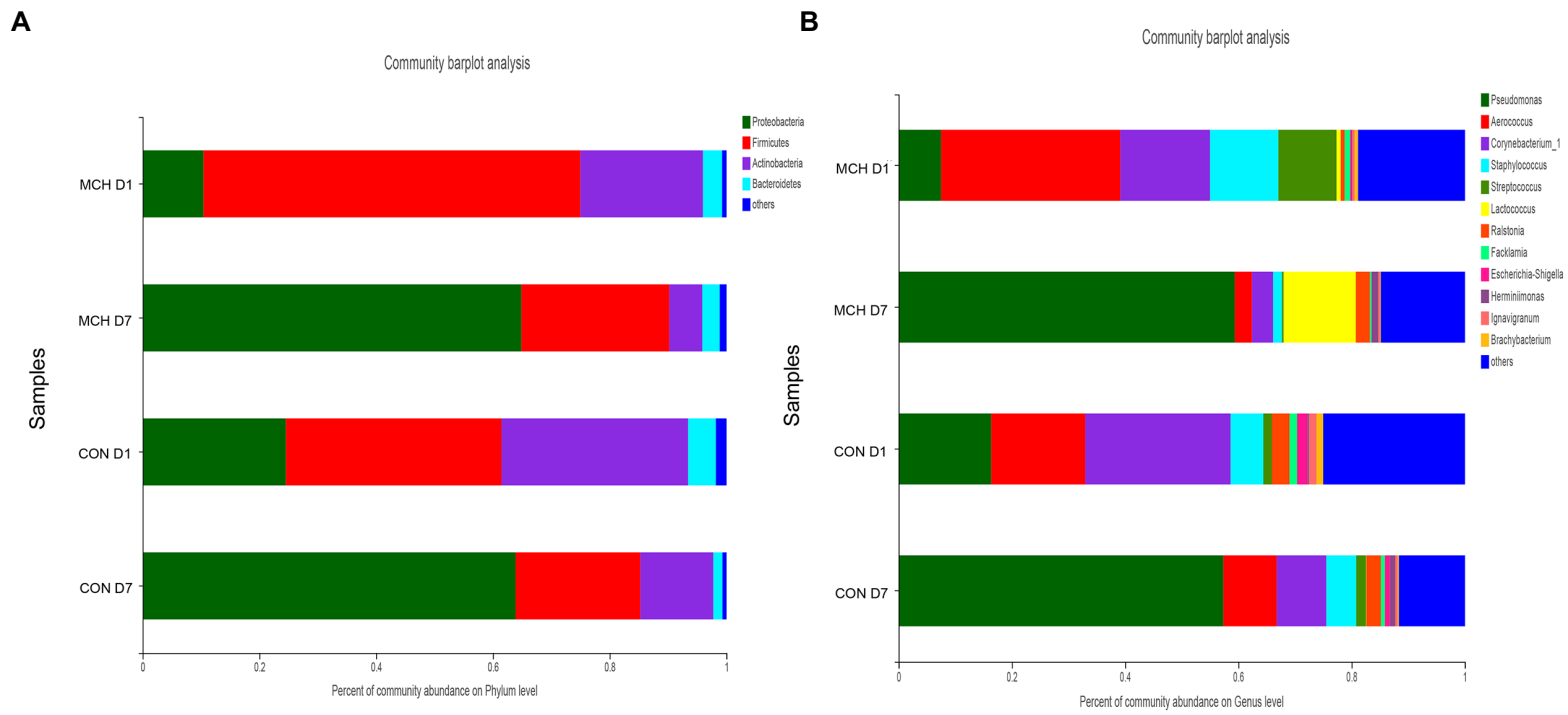
Classification of the bacterial community composition in milk samples on day 1 and day 7 between MCH group and CON group, *n* = 6. **(A)** Phylum level. **(B)** Genus’s level.

**Figure 3:**
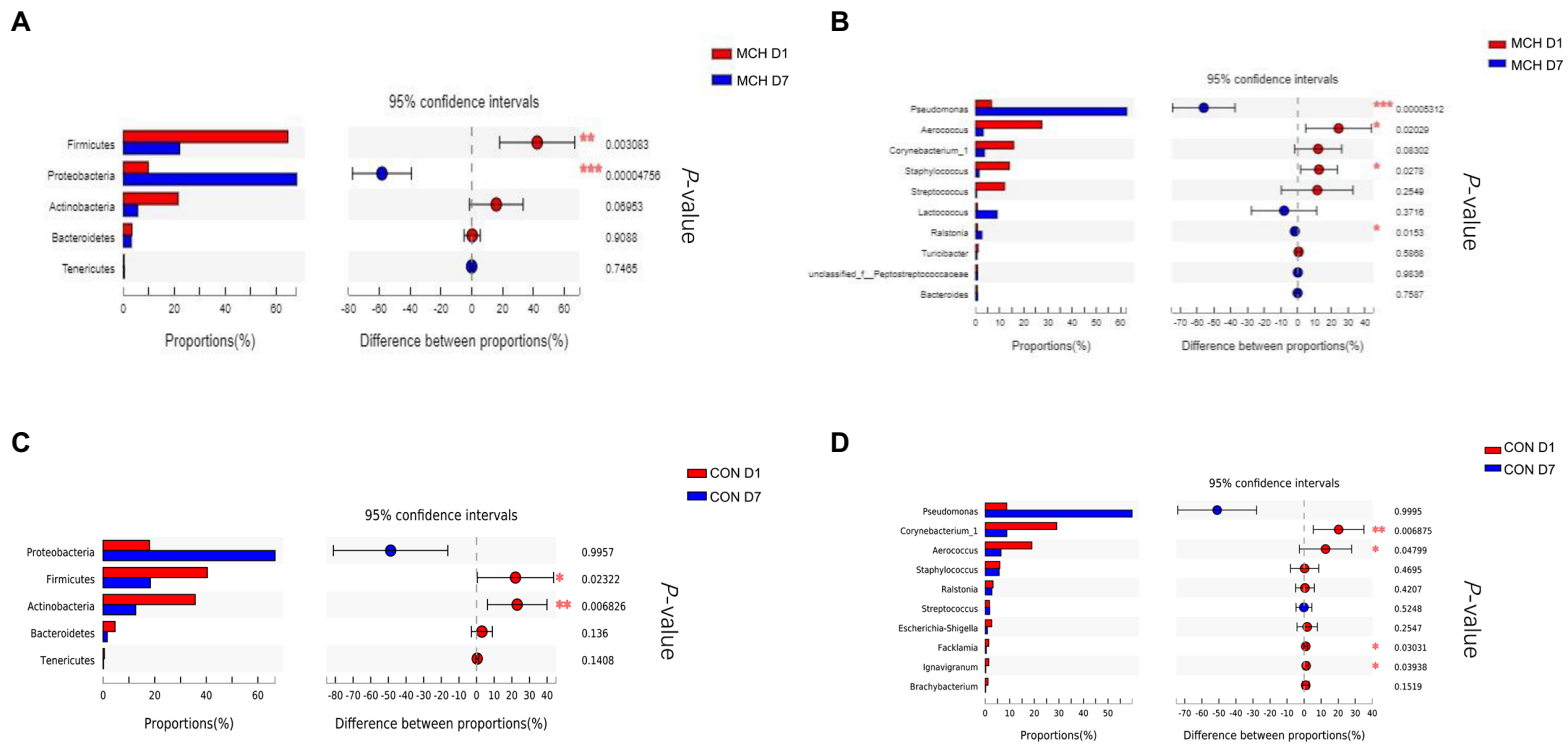
Differences in relative abundance of the main bacterial in milk samples on day 1 and day 7 between MCH group and CON group, *n* = 6. **(A)** MCH main bacterial phyla. **(B)** MCH main bacterial genera. **(C)** CON main bacterial phyla. **(D)** CON main bacterial genera.

In MCH group, the taxonomic changes in milk samples from day 1 to day 7 were determined. *Proteobacteria, Firmicutes, Actinobacteria* and *Bacteroidetes* were the three predominant phyla (Figure 2A). Taxa with a relative abundance of 1 % in at least one sample were identified, and the 10 most abundant genera are presented (Figure 2B). The abundance of *Firmicutes* was extremely significantly decreased on day 7 (*p* = 0.01), whereas the numbers of *Proteobacteria* increased (*p* = 0.01; Figure 3A). On day 7, there was a significantly lower abundance of *Aerococcus* (*p* = 0.01), *Corynebacterium_1* (*p* = 0.08) and *Staphylococcus* (*p* = 0.03). In contrast, the relative abundances of *Pseudomonas* (*p* = 0.01) and *Ralstonia* (*p* = 0.02) were significantly increased (Figure 3B). Meanwhile, the same tendency was observed in the CON group (Figures 3C,D).

### Identification and comparison of milk metabolites

A non-targeted metabolome method was used to evaluate milk metabolites after MCHs and ceftiofur hydrochloride treatment. Reproducible metabolite data profiles were obtained and differences between two groups on D1 and D7 were characterized using VIP metrics from OPLS-DA. Seventy-four milk metabolite signals were obtained that were significantly different between MCH-treated and untreated cows (VIP >1 and *p* < 0.05; Table 4), the CON group milk metabolite in Supplementary Table 1. They consisted mainly of sphingolipids, oxanes, glycerophospholipids, flavonoids, and fatty acyls. Milk from animals receiving MCHs for 7 days had higher levels of steroids and steroid derivatives, prenol lipids, oxanes, macrolides and analogues, hydroxy acids and derivatives, carboxylic acids and derivatives, benzene, and substituted derivatives than milk on D1.

**Table 4:** Milk metabolites on day 1 compared to day 7 of MCH treatment (*n* = 6).

**Metabolite**	**M/Z**	**Retention time**	**Mass error**	**VIP**	**FC(d1/d7)**	***P*-value**	**Trend**
Steroids and steroid derivatives							
12b-hydroxy-5b-cholanoic acid	418.35	9.43	1.12	4.42	0.48	0.01	↑
Pregnanetriolone	349.24	8.8	−0.06	1.79	0.43	0.026	↑
Aginoside progenin	814.46	2.51	−2.8	1.02	0.04	<0.01	↑
Sphingolipids							
SM(d18:0/24:1(15Z))	859.7	13.36	5.06	1.58	3,208,275,862	<0.01	↓
SM(d18:1/23:0)	845.68	12.95	5.27	2.26	11748.6	<0.01	↓
SM(d18:1/22:1(13Z))	829.65	11.92	4.59	1.72	369.77	<0.01	↓
SM(d18:0/22:1(13Z))	831.66	12.73	4.91	4.9	212.45	<0.01	↓
Glucosylceramide (d18:1/16:0)	744.57	11	3.44	1.89	34.01	<0.01	↓
Galactosylceramide (d18:1/14:0)	706.51	10.5	3.07	1.29	27.94	<0.01	↓
Glucosylceramide (d18:1/25:0)	790.69	14.01	−0.89	3.78	21.09	<0.01	↓
stearoyl sphingomyelin	775.6	11.48	4.77	1.32	19.61	<0.01	↓
N-hexadecanoylsphinganine-1-phosphocholine	749.58	11.16	2.57	2.76	16.13	<0.01	↓
Galabiosylceramide (d18:1/16:0)	906.62	10.86	5.8	1.9	9.04	<0.01	↓
SM(d18:0/14:0)	721.55	10.62	3.35	2.94	7.99	<0.01	↓
SM(d18:0/16:1(9Z))	747.57	10.92	4.52	6.89	3.62	<0.01	↓
N-Glycoloylganglioside GM2	708.61	12.75	−0.87	9.56	2.38	<0.01	↓
SM(d18:1/14:0)	719.54	10.42	3.7	5.12	2.34	<0.01	↓
Araliacerebroside	776.55	10.75	−6.85	1.95	1.49	<0.01	↓
Ganglioside GM1 (d18:1/18:1(11Z))	772.43	4.33	−7.63	1	0.28	0.02	↑
nLc6Cer	696.82	3.25	−1.78	2.36	0.09	<0.01	↑
Prenol lipids							
Hoduloside VI	819.43	2.96	−3.62	1.42	0.05	0.01	↑
Oxanes							
D-1,5-anhydrofructose	325.11	0.78	−0.78	1.1	0.78	<0.01	↑
Organo-oxygen compounds							
3,4,5-trihydroxy-6-(2-hydroxyethoxy) oxane-2-carboxylic acid	475.13	0.7	−1.46	1.13	2.62	<0.01	↓
N6-galacturonyl-L-lysine	357.11	2.4	8.9	2.54	1.56	0.02	↓
Lactulose	365.11	0.62	1.53	6.7	0.56	<0.01	↑
Macrolides and analogues							
Pectenotoxin 7	909.37	2.45	−1.88	1.95	0.1	<0.01	↑
31-O-demethyltacrolimus	810.44	0.75	−1.37	1.15	0.03	<0.01	↑
Indoles and derivatives							
Indole-3-carboxilic acid-O-sulfate	240	2.27	−1	3.13	1.74	<0.01	↓
Hydroxy acids and derivatives							
2-hydroxy-3-methoxyestrone	442.35	9.08	1.05	2.9	0.43	0.01	↑
Glycerophospholipids							
PE (15:0/22:0)	806.6	11.84	5.06	2.5	48.31	<0.01	↓
PE (14:0/22:0)	792.58	11.45	8.49	1.14	15.03	<0.01	↓
PS (DiMe (11,3)/MonoMe (11,5))	880.54	12.16	2.74	1.21	8.01	<0.01	↓
PE (15:0/24:1(15Z))	832.61	11.77	5.01	4.77	6.11	<0.01	↓
lysoPC (6:0)	400.17	3.01	−1.21	2.04	5.51	<0.01	↓
PE (16:0/16:0)	736.52	10.42	2.96	2.16	2.26	<0.01	↓
PS (18:0/18:1(9Z))	810.53	11.5	5.99	2.68	2.12	<0.01	↓
PE (18:2(9Z,12Z)/18:1(11Z))	786.53	12.04	4.76	7.92	1.99	<0.01	↓
PE (15:0/16:0)	722.5	10.33	5.04	3.2	1.85	<0.01	↓
PE (15:0/14:0)	694.47	9.94	3.99	1.33	1.72	<0.01	↓
PE (15:0/22:1(13Z))	804.58	11.24	5.01	4	1.67	<0.01	↓
PS (16:0/18:0)	784.52	11.45	7.51	3.58	1.63	<0.01	↓
LysoPE (0:0/18:2(9Z,12Z))	476.28	7.75	−0.17	1.66	1.53	0.05	↓
PE (18:1(9Z)/16:0)	718.54	11.44	0.48	1.83	1.5	0.03	↓
PE (15:0/20:2(11Z,14Z))	774.53	10.37	4.68	1.03	1.39	0.02	↓
LysoPE (16:0/0:0)	452.28	8.32	0.26	1.84	1.36	0.01	↓
PS (15:0/24:1(15Z))	830.6	11.21	4.84	1.71	1.3	0.02	↓
LysoPC (18:1(9Z))	566.35	8.31	0.06	3.94	0.68	0.01	↑
LysoPC (18:2(9Z,12Z))	564.33	7.79	−1.16	5.06	0.56	0.01	↑
LysoPC (16:1(9Z)/0:0)	538.32	7.58	1.76	1.12	0.55	0.02	↑
LysoPE (20:3(11Z,14Z,17Z)/0:0)	502.29	8.11	0.14	1.65	0.5	0.02	↑
PC (18:2(9Z,12Z)/P-18:0)	814.6	11.07	4.43	1.18	0.47	0.01	↑
LysoPE (20:4(5Z,8Z,11Z,14Z)/0:0)	500.28	7.8	−0.15	3.45	0.41	<0.01	↑
LysoPE (18:2(9Z,12Z)/0:0)	478.29	7.77	2	2.75	0.35	<0.01	↑
LysoPC (18:1(11Z))	522.36	8.2	0.69	4.22	0.31	<0.01	↑
LysoPE (0:0/22:5(7Z,10Z,13Z,16Z,19Z))	562.27	5.39	−0.86	1.06	0.3	<0.01	↑
1-Linoleoylglycerophosphocholine	520.34	7.69	0.61	4.27	0.17	0.01	↑
1-(8Z,11Z,14Z-eicosatrienoyl)-glycero-3-phosphate	502.29	7.7	−0.01	1.27	0.14	<0.01	↑
LysoPC (P-18:0)	552.37	8.59	0.37	1.31	0.09	<0.01	↑
TG (16:1(9Z)/16:1(9Z)/16:1(9Z))	845.68	13.12	−9.94	3.56	1607.82	<0.01	↓
MG (0:0/16:0/0:0)	365.25	8.94	−0.54	3.43	0.53	0.027	↑
Flavonoids							
Menthosides	723.2	0.7	4.79	4.57	2.69	<0.01	↓
Isoscoparin 2′-(6-(E)-ferulylglucoside)	781.2	0.84	−0.3	2.37	1.31	0.02	↓
Kaempferol 3-(2′-rhamnosylgalactoside) 7-rhamnosides	777.16	0.7	−1.93	4.24	0.49	<0.01	↑
Licorice glycoside C1	765.18	0.64	0.82	2.27	0.19	<0.01	↑
Fatty Acyls							
cis-uvariamicin IB	627.48	11.55	2.27	1.32	1268.67	<0.01	↓
3,4-dimethyl-5-pentyl-2-furanpentadecanoic acid	857.68	12.57	−9.88	1.54	130.24	<0.01	↓
15-hydroxyeicosanoic acid	698.63	13.78	−0.76	4.76	11.33	<0.01	↓
2-hydroxyhexadecanoic acid	271.23	8.73	0.84	2.08	0.47	0.01	↑
3-hydroxyhexadecanoyl carnitine	416.34	8.99	2.02	2.15	0.43	0.01	↑
Aminocaproic acid	132.1	1.17	0.75	1.42	0.04	<0.01	↑
Carboxylic acids and derivatives							
Tyrosyl-Isoleucine	293.15	2.31	−0.33	1.53	0.22	<0.01	↑
L-Phenylalanine	164.07	1.71	0.73	1.03	0.09	<0.01	↑
Benzene and substituted derivatives							
fluvoxamino acid	360.15	0.86	−8.35	2.72	0.53	<0.01	↑

**Figure 4:**
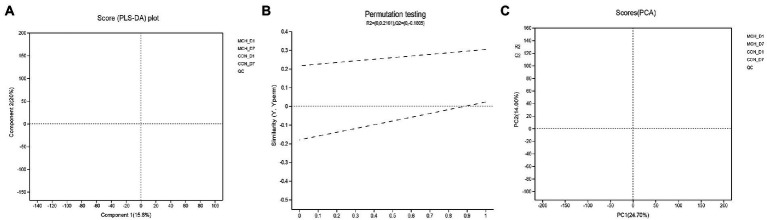
Principal component analysis score plot **(A)**, permutation test plot **(B)** and orthogonal partial least squares discriminant analysis (OPLS-DA) **(C)** for days 1 and 7 between MCH group and CON group based on milk metabolite profiles. The variation in the principal components is indicated on the axes. Each spot is one sample, and the days are shown as green circles for day 1 and blue triangles for day 7 (*n* = 6).

PCA and OPLS-DA were employed to evaluate the differences in metabolomes. The PCA scores showed clear differences between D1 and D7 milk samples (Figure 4A). Principal components one and two were found to account for 15.6 and 20% of the variation, respectively. The variables for assessing OPLS-DA model quality are shown as validation plots (Figure 4B). The Q2 value of the OPLS-DA model was −0.18, and the R2Y value was 0.21. Q2 represents a model’s prediction ability and the closer these three indicators are to 1, the more stable and reliable it is. The OPLS-DA data confirmed that the two groups had significantly different types and levels of metabolites (Figure 4C). OPLS-DA model integrity is shown by validation plots. The D7 metabolite profiles were significantly different from those of D1 indicating that the PCA and OPLS-DA results were valid for assessing variations in milk metabolomes between the 2 days.

### Differences in metabolites resulting from changes in metabolic pathways

**Figure 5:**
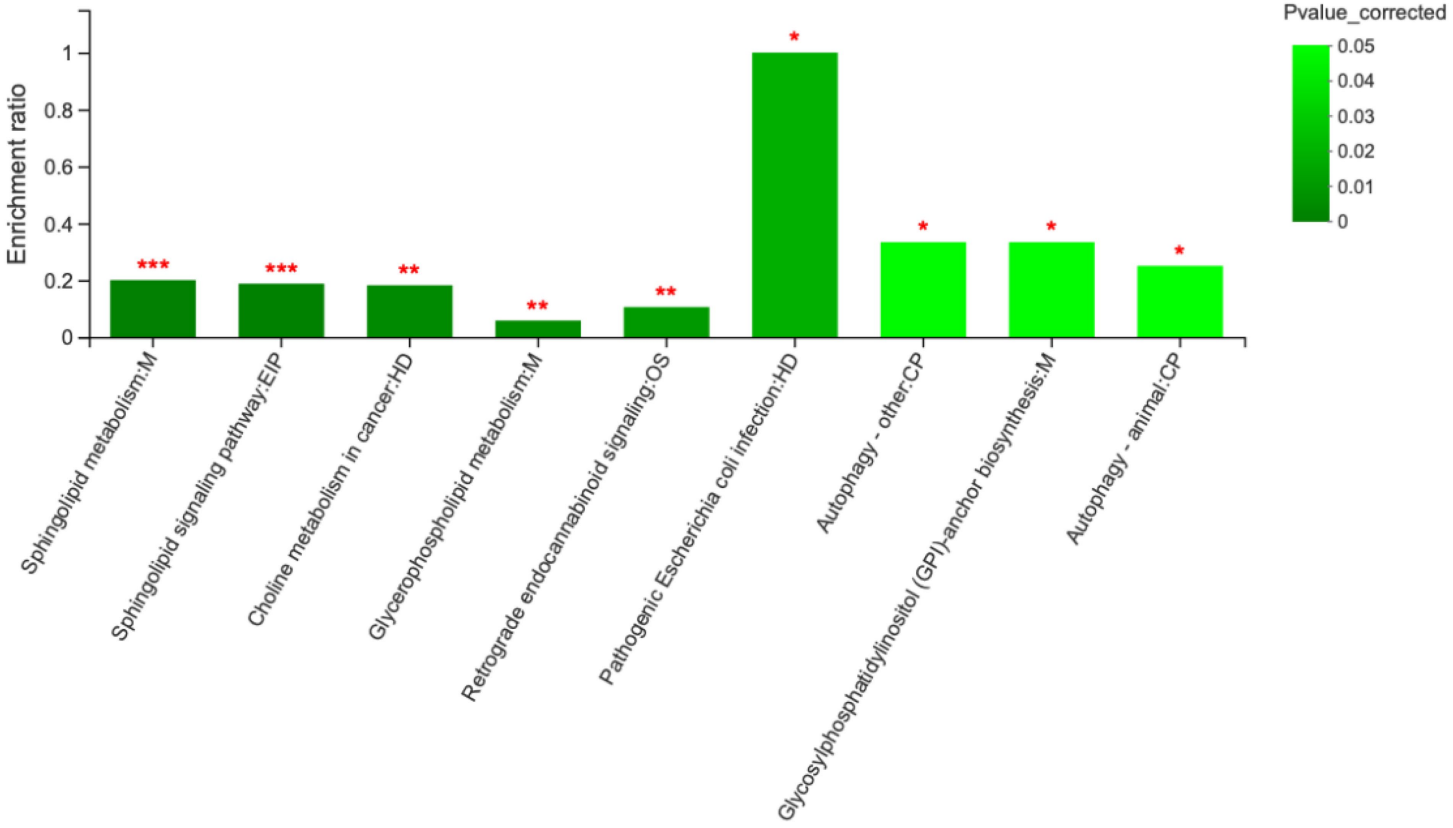
Metabolic pathway enrichment analysis between day 1 and day 7. M, EIP, HD, OS and CP are the names of the metabolic pathways in KEGG annotation. M, metabolism; EIP, environmental information processing; HD, human diseases; OS, organismal systems; CP, cellular processes (*n* = 6). ***p < 0.001, **p < 0.01, and *p < 0.05.

**Figure 6:**
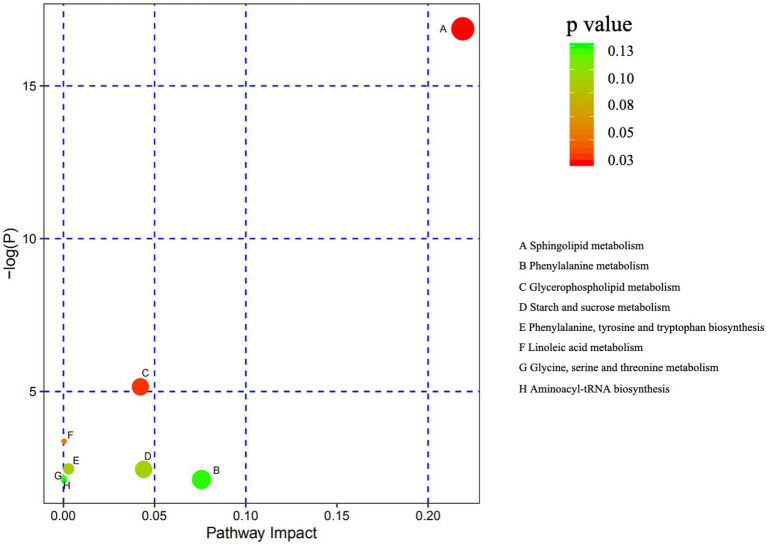
Metabolome mapping of the differences in metabolite expression from day 1 to day 7. The abscissa shows pathway impact and the ordinate gives the *p* value. The bigger the circles the greater the number of metabolites enriched in the pathway. Darker colors indicate smaller *p* values (*n* = 6).

Comparison of metabolite levels relative to changes in pathway activation from MCHs treatment on D1 to D7 was performed using the Kyoto Encyclopedia of Genes and Genomes (KEGG) for determining enrichment of pathways involved in metabolite production (Table 5; Figure 5). Enrichment analysis showed that sphingolipid metabolism, the sphingolipid signaling pathway, glycerophospholipid metabolism and ABC transporters, were significantly affected by MCHs infusion. Pathway topology analysis (Figure 6; *p* < 0.05) revealed eight key pathways enriched between days 1 and 7. They included sphingolipid, phenylalanine, glycerophospholipid, starch and sucrose metabolism, synthesis of phenylalanine, tyrosine and tryptophan and linoleic acid metabolism. For the CON group, the enrichment of pathways analysis was shown in Supplementary Figure 1.

**Table 5:** Differences in metabolites enriched from specific pathways in milk of dairy cows receiving MCH infusions (7 days, *n* = 6).

**Metabolic pathway**	**Metabolite**
Sphingolipid metabolism (10)	Galabiosylceramide (d18:1/16:0); SM(d18:1/22:1(13Z)); SM(d18:0/22:1(13Z)); Glucosylceramide (d18:1/16:0); SM(d18:0/16:1(9Z)); Glucosylceramide (d18:1/25:0); SM(d18:0/24:1(15Z)); Galactosylceramide (d18:1/14:0); SM(d18:0/14:0); SM(d18:1/23:0)
Sphingolipid signaling pathway (6)	SM(d18:1/22:1(13Z)); SM(d18:0/22:1(13Z)); SM(d18:0/16:1(9Z)); SM(d18:0/24:1(15Z)); SM(d18:0/14:0); SM(d18:1/23:0)
Glycerophospholipid metabolism (3)	PE(14:0/24:1(15Z)); LysoPC(20:4(8Z,11Z,14Z,17Z)); PC(15:0/16:0)
Choline metabolism in cancer (2)	LysoPC(20:4(8Z,11Z,14Z,17Z));PC(15:0/16:0)
Retrograde endocannabinoid signaling (2)	PE (14:0/24:1(15Z)); PC (15:0/16:0)
Pathogenic Escherichia coli infection (1)	PE (14:0/24:1(15Z))
Autophagy – other (1)	PE (14:0/24:1(15Z))
Glycosylphosphatidylinositol (GPI)-anchor biosynthesis (1)	PE (14:0/24:1(15Z))
Autophagy – animal (1)	PE (14:0/24:1(15Z))

## Discussion

The results of this study revealed that matrine-chitosan hydrogel markedly decreased SCCs after 7 days of treatment. Although we did not observe any changes in bacterial community richness by the Chao indices, the Shannon index showed a significant decrease in bacterial diversity. On D7, the relative abundance of Firmicutes was significantly decreased, and that of Proteobacteria was significantly increased compared to levels on D1. It has been reported that the richness and diversity of the milk microbiome reflects the health of cows and the functional performance of their organs (Mohammed et al., 2014; Schokker et al., 2015; Blanco et al., 2016). Matrine has antibacterial and anti-inflammatory properties and has been commonly used to treat bovine mastitis, but its efficacy is still unproven (Oliveira and Ruegg, 2014). That is why we chose to test the plant-derived compound, matrine, as an intramammary treatment loaded into chitosan hydrogels to see if it would alter the milk microbiota and improve mammary gland function.

Measurement of the number of somatic cells in milk is an internationally recognized test for the detection of mastitis. A SCC of more than 200,000 per ml is evidence of disease (Kovac et al., 2007). Recent investigations have shown that the milk microbiota of dairy cows is usually closely associated with the SCC (Oikonomou et al., 2014; Vanderhaeghen et al., 2014). This suggests that modulation of the udder ecosystem through the microbiota could help to maintain homeostasis and enhance the mammary gland’s defenses. Consistent with these reports, we observed that the udder SCC was significantly increased in mastitis caused by common pathogens like staphylococci, but was markedly decreased after perfusion of the udder with matrine-chitosan hydrogel. This is in line with previous studies showing that chitosan hydrogels decreased the SCC in milk from dairy cows (Lanct et al., 2017). The therapeutic synergy achieved by loading the chitosan hydrogel with matrine suggests potential applications in mastitis protection and treatment. The mechanism by which the milk microbiota influences inflammatory and immune responses to decrease the SCC needs further investigation.

The next generation of high-throughput DNA sequencing methods together with updated bioinformatics software is now being used for the in-depth assessment of microbial communities to elucidate how bacterial activity and metabolites affect human and animal health (Catozzi et al., 2017). We found that the microbial diversity in milk was dramatically decreased after 1 week of intramammary infusion of matrine-chitosan hydrogels. The relative abundance of Proteobacteria was significantly increased, while staphylococcus decreased significantly. Staphylococci are recognized as one of the most prevalent mastitis pathogens, accounting for about 70% of the cases (Gao et al., 2017). Moreover, it has been reported that Proteobacteria were significantly more abundant in healthy cows than in those with mastitis (Catozzi et al., 2017). Previous studies also showed that the numbers of Proteobacteria after non-antibiotic hydrogel treatment increased significantly (Bhatt, 2012), indicating that hydrogel therapy can effectively return the milk microbial environment to normal diversity and defend against disease. Our investigation provides mechanistic insights into how the microbiota respond to MCHs that may help in the development of novel prophylactic and therapeutic products as alternatives to antibiotics in dairy cows. Our data are also important for understanding how the regulation of biosynthesis by milk microbiota influences udder health and defense against mastitis. The milk microbiota is usually closely associated with the SCC (Oikonomou et al., 2014; Vanderhaeghen et al., 2014) suggesting that the udder microbiota is important in modulation of the udder ecosystem, maintenance of homeostasis, and resistance of the mammary gland to infections (Barkema et al., 2006; Rainard and Pascal, 2017).

Metabolomics is a comparatively new research method that has been widely used in the detection of mastitis in recent years due to its more comprehensive test results. Previous studies suggested that metabolomics could provide a more complete understanding of an animal’s physiology and biochemistry (Zhao et al., 2014). The metabolomics data from this study highlight the potential function of matrine-chitosan hydrogels in modulating the metabolite levels, which were enriched in sphingolipid metabolic pathways, phenylalanine, glycerophospholipid, and starch and sucrose metabolism. A previous report suggested that sphingolipid metabolites acted as signaling molecules to regulate a diverse group of cellular processes, particularly those related to immunity, inflammation, and inflammatory disorders (Maceyka and Spiegel, 2014). We speculate that this activity might be associated with the decrease in SCC caused by infusion of matrine-chitosan hydrogels. The metabolic differences we observed gave us further insights into how MCHs affects metabolite levels after mastitis treatment. The data showed that alterations in the milk metabolome could be used to reveal the therapeutic mechanism of matrine therapy for mastitis treatment and in the recovery of milk production in dairy cows.

We found significant changes in the concentrations of sphingolipids, glycerolipids, fatty acyls, glycerophospholipids, and organo-oxygen compounds from D1 to D7. Sphingolipids are major components of cell membranes and are widely involved in important processes such as cell aging and apoptosis (Thomas et al., 2016). Glycerolipids (Li N. et al., 2018) and fatty acyls (Kagan et al., 2017) are both important components of cell membranes. Previous research found that bacterial invasion could induce inflammatory reactions accompanied by apoptosis and cause oxidative damage during breast development (Turk, 2017). The reduction in sphingolipids, glycerolipids, and fatty acyls seen in this study indicates that apoptosis caused by inflammatory reactions may have been lessened. The decrease of organo-oxygen compounds suggests that the oxidative damage caused by inflammatory reactions may also have been reduced. The metabolites were enriched in sphingolipid and glycerophospholipids, which are both related to apoptotic pathways (Gao et al., 2016, 2018). We conclude that MCHs can inhibit apoptosis by blocking oxidative damage, thereby reducing inflammatory changes to the udder.

It is worth noting that L-phenylalanine was involved in many pathways that were significantly changed in this study (Table 3). L-phenylalanine is an essential amino acid as well as a precursor for the commercial synthesis of antiviral and anticancer drugs. It can also be a marker of inflammatory reactions and affect the body’s immune response (Turk, 2017). Here, the content of L-phenylalanine was significantly increased on day 7, which means that the immune defenses in the mammary gland may have been effectively strengthened. In a previous study, Strasser et al. (2016) found that tryptophan and phenylalanine were used in the body to synthesize serotonin, L-DOPA derivatives and 5, 6, 7, 8-tetrahydrobiopterin (BH4), which compounds can affect the severity of inflammation. In the study of Smith et al. (2018), tyrosine and phenylalanine participated in the regulation of TLR4 signaling pathways, thereby influencing the degree of inflammation. Phenylalanine also is part of the glycolytic and liposynthetic pathways (Waisbren et al., 2007; Obianom et al., 2019). It has been reported that phenylalanine deficiency can severely affect protein metabolism in the breast and compromise the health of the organ (Shibata et al., 1992). Phenylalanine is an essential amino acid in milk and is also the precursor of tyrosine, which is one of the main amino acids in milk (Rezaei et al., 2016). We know that untreated bovine mastitis results in reduced milk fat and milk protein content; therefore, the increase in L-phenylalanine by MCHs infusion could be effective in restoring normal milk production during lactation.

The milk metabolites induced by MCHs were found to have an intimate relationship with sphingolipid and glycerophospholipid metabolism. A previous study suggested that the sphingolipid metabolic pathways participate in a variety of immune-related signal transductions, inflammation, and inflammatory disorders (Maceyka and Spiegel, 2014). The sphingolipid metabolic pathways have many functions, such as regulating cell adhesion and cellular immunity, activating cancer repressors, regulating apoptosis, and modulating immune function and the inflammatory response (Maceyka and Spiegel, 2014). We speculate that this activity might be associated with the decrease in SCC caused by MCHs. Brice and Cowart (2011) showed that an imbalance in the pathway for sphingolipid metabolism caused ketosis, mastitis, and metritis in cows. It has been proved that sphingolipid metabolites, especially ceramide and sphingosine-1-phosphate, can regulate a variety of biochemical processes important in immunological and inflammatory diseases (Maceyka and Spiegel, 2014). In the present study, we found that MCHs significantly regulated sphingolipid pathways to reduce inflammation; thus, it is reasonable to believe that MCHs could effectively treat bovine mastitis.

Despite the documented health-promoting properties of matrine and chitosan, scientific evidence for the efficacy of MCHs in dairy cows is limited. Our study affords many useful insights, but the mechanism of MCH’s effect on immune regulation still requires further study. Another limitation relates to the mechanistic links involved in the observed significant changes in the microbiota and biomarker metabolites as a result of MCHs infusion that need further exploration to be clearly understood. Previous studies suggested that the antimicrobial activity of chitosan stimulated the innate immune response and hastened involution of the mammary gland (Lanct et al., 2017). It is not known whether matrine-chitosan complexes could be used in the circulation to regulate the immune system and inhibit inflammation, but our data indicate that future research in this area is strongly warranted.

## Conclusion

Our data indicate that matrine-chitosan hydrogels significantly decrease the somatic cell counts and affect the structures of bacterial communities in the udder, especially the relative abundances of *Corynebacterium*_1, *Aerococcus,* and *Staphylococcus*. Our findings show significant changes in metabolites and metabolic pathways as a result of intramammary MCHs infusion, and some of the 74 resulting milk metabolites may be used as indicators of the response to MCHs treatment. The results from the milk metabolic pathway analysis are promising for the investigation of matrine-chitosan’s antimicrobial and anti-inflammatory properties that are closely associated with sphingolipid and glycerophospholipid metabolism. These insights into the complex mechanisms and corresponding biological responses highlight the beneficial action of matrine-chitosan hydrogels and justify continued investigations to identify the immunoregulatory mechanisms for treatment of mastitis in dairy cows.

## Data availability statement

The datasets presented in this study can be found in online repositories. The names of the repository/repositories and accession number(s) can be found at: NCBI-SRP254162.

## Ethics statement

The animal study was reviewed and approved by Animal Care Committee of Beijing University of Agriculture.

## Author contributions

JT, ZW, and LJ developed hypothesis, conceived the project, and responsible for all data, figures, and text. JT, HZ, and ZW performed the experiments. ZW and HY conducted data analysis. JT and ZW wrote the manuscript. JT, HZ, and LJ revised the paper. All authors contributed to the article and approved the submitted version.

## Funding

This study was funded by the Project of the National Natural Science Foundation of China (Grant No. 31802091). JT thanks the Beijing University Youth Talent Science Foundation and R&D Program of Beijing Municipal Education Commission (KM202210020006).

## Conflict of interest

The authors declare that the research was conducted in the absence of any commercial or financial relationships that could be construed as a potential conflict of interest.

## Publisher’s note

All claims expressed in this article are solely those of the authors and do not necessarily represent those of their affiliated organizations, or those of the publisher, the editors and the reviewers. Any product that may be evaluated in this article, or claim that may be made by its manufacturer, is not guaranteed or endorsed by the publisher.

## Footnotes

^1^ https://metlin.scripps.edu/landing_page.php?pgcontent=mainPage

^2^ https://cloud.majorbio.com

^3^ https://www.metaboanalyst.ca

## Abbreviations

MCHs: Matrine-chitosan hydrogels
PCA: Principal component analysis
OPLS-DA: Orthogonal partial least squares discriminant analysis
KEGG: Kyoto Encyclopedia of Genes and Genomes
M: Metabolism
HD: Human diseases
EIP: Environmental information processing
OS: Organismal systems
GIP: Genetic information processing
SCC: Somatic cell counts
CMT: California mastitis rapid detection reagent
RDP: Ribosomal database project
OTUs: Operational taxonomic units
