# Supplementary material for: Intramammary infusion of matrine-chitosan hydrogels for treating subclinical bovine mastitis —effects on milk microbiome and metabolites
Source: Front Microbiol. 2022 Sep 20;13:950231. doi: 10.3389/fmicb.2022.950231 (PMC9530655; doi:10.3389/fmicb.2022.950231)
Supplement: Supplementary file 1 [file Data_Sheet_1.docx]

**Supplementary Fig 1.** Metabolic pathway enrichment analysis on CON group between day 1 and day 7. M, EIP, HD, OS and CP are the names of the metabolic pathways in KEGG annotation. M, metabolism; EIP, environmental information processing; HD, human diseases; OS, organismal systems; CP, cellular processes (n=6).

**Supplementary Fig 2.** Metabolome mapping of the differences in metabolite expression from day 1 to day 7 of CON group . The abscissa shows pathway impact and the ordinate gives the *P* value. The bigger the circles the greater the number of metabolites enriched in the pathway. Darker colors indicate smaller *P* values (n = 6).

**Supplementary Table 1.** Milk metabolites on day 1 compared to day 7 of CON treatment (n = 6)

| **Metabolite** | **M/Z** | **Retention** | **VIP** | **FC(d1/d7)** | ***P* value** | | **Trend** |
| --- | --- | --- | --- | --- | --- | --- | --- |
|  |  | **time** |  |  |  |  |  |
| Ile Trp | 318.18 | 2.99 | 1.67 | 0.33 | 0.03 |  | ↑ |
| N(6)-(Octanoyl)lysine | 586.45 | 7.52 | 3.84 | 0.22 | 0.03 |  | ↑ |
| 3,4-Dimethyl-5-pentyl-2-furanpropanoic acid | 271.19 | 10.67 | 0.97 | 5.36 | 0.04 |  | ↓ |
| Glucosylceramide (d18:1/20:0) | 720.61 | 12.38 | 1.11 | 3.61 | 0.03 |  | ↓ |
| 15-hydroxyicosanoic acid | 698.63 | 13.78 | 2.77 | 2.92 | 0.04 |  | ↓ |
| 12-Ketodeoxycholic acid | 355.26 | 6.03 | 1.48 | 0.28 | 0.05 |  | ↓ |
| Dihydroceramide | 362.33 | 5.84 | 1.50 | 0.62 | 0.02 |  | ↓ |
| PS(20:3(5Z,8Z,11Z)/22:2(13Z,16Z)) | 907.62 | 4.53 | 0.94 | 0.23 | 0.02 |  | ↓ |
| Galactosylceramide (d18:1/14:0) | 706.51 | 10.50 | 1.69 | 5.86 | 0.03 |  | ↓ |
| PE(14:0/24:1(15Z)) | 818.60 | 11.51 | 0.95 | 2.65 | 0.04 |  | ↓ |
| Cer(d18:0/16:0) | 574.50 | 11.55 | 1.03 | 11.49 | 0.04 |  | ↓ |
| SM(d18:0/18:0) | 777.62 | 11.71 | 1.16 | 10.25 | 0.04 |  | ↓ |
| SM(d18:0/22:0) | 833.68 | 13.03 | 1.46 | 31.46 | 0.04 |  | ↓ |
| Glucosylceramide | 846.66 | 13.56 | 1.49 | 41.86 | 0.02 |  | ↓ |
| TG(16:1(9Z)/16:1(9Z)/16:1(9Z)) | 845.68 | 13.12 | 2.61 | 8.64 | 0.05 |  | ↓ |
| N-Stearoylsphingosine | 600.51 | 11.92 | 0.84 | 10.22 | 0.03 |  | ↓ |
| Orotic acid | 155.01 | 0.79 | 1.75 | 1.76 | 0.02 |  | ↓ |
| (3-phenylpropoxy)sulfonic acid | 215.04 | 4.09 | 0.82 | 2.85 | 0.04 |  | ↓ |
| Glucosylceramide (d18:1/16:0) | 744.57 | 11.00 | 2.19 | 6.27 | 0.03 |  | ↓ |
| PC(18:1(11Z)/P-16:0) | 788.58 | 11.49 | 1.15 | 3.48 | 0.02 |  | ↓ |
| SM(d18:0/22:1(13Z)) | 831.66 | 12.73 | 3.26 | 7.32 | 0.04 |  | ↓ |
| 3,4-Dimethyl-5-pentyl-2-furanpentadecanoic acid | 857.68 | 12.57 | 1.83 | 7.12 | 0.04 |  | ↓ |
| SM(d16:1/24:1(15Z)) | 820.62 | 12.06 | 1.74 | 11.60 | 0.02 |  | ↓ |
| SM(d18:1/22:1(13Z)) | 829.65 | 11.92 | 2.21 | 9.39 | 0.02 |  | ↓ |
| PE(14:0/22:0) | 792.58 | 11.45 | 1.65 | 3.62 | 0.05 |  | ↓ |
| PC(15:0/16:0) | 754.52 | 11.02 | 0.92 | 3.40 | 0.05 |  | ↓ |
| AS 1-5 | 760.56 | 10.90 | 1.18 | 2.41 | 0.03 |  | ↓ |
| PE(16:0/20:2(11Z,14Z)) | 788.55 | 10.58 | 2.38 | 1.28 | 0.05 |  | ↓ |
| LysoPE(20:4(5Z,8Z,11Z,14Z)/0:0) | 500.28 | 7.80 | 2.96 | 0.56 | 0.04 |  | ↓ |
| 2-Hydroxyestrone | 617.31 | 2.27 | 0.86 | 0.27 | 0.05 |  | ↓ |
| Isoscoparin 2''-(6-(E)-ferulylglucoside) | 781.20 | 0.84 | 2.78 | 1.23 | 0.04 |  | ↓ |
| Oxoglutaric acid | 145.01 | 0.71 | 1.29 | 2.91 | 0.04 |  | ↓ |

**Supplementary Table 2.** Differences in metabolites enriched from specific pathways in milk of dairy cows receiving CON infusions (7 days ，n = 6).

| **Metabolic Pathway** | **Metabolite** |
| --- | --- |
| Sphingolipid signaling pathway (6) | Dihydroceramide; SM(d18:0/18:0); SM(d18:0/22:0); N-Stearoylsphingosine; SM(d18:0/22:1(13Z)); SM(d18:1/22:1(13Z)) |
| Sphingolipid metabolism (10) | Glucosylceramide (d18:1/20:0);Dihydroceramide; Galactosylceramide (d18:1/14:0); SM(d18:0/18:0);SM(d18:0/22:0);Glucosylceramide;N-Stearo ylsphingosine;Glucosylceramide (d18:1/16:0);SM(d18:0/22:1(13Z));SM(d18:1/22:1(13Z)) |
| Retrograde endocannabinoid signaling (2) | PE(14:0/24:1(15Z)); PC(15:0/16:0) |
| Neurotrophin signaling pathway(1) | N-Stearoylsphingosine |
| Leishmaniasis(1) | N-Stearoylsphingosine |
| Glycosylphosphatidylinositol (GPI)-anchor biosynthesis (1) | PE(14:0/24:1(15Z)) |
| Glycerophospholipid metabolism (2) | PE(14:0/24:1(15Z)); PC(15:0/16:0) |
| Autophagy – other (1) | PE(14:0/24:1(15Z)) |
| Autophagy – animal (1) | PE(14:0/24:1(15Z)) |
| Adipocytokine signaling pathway(1) | N-Stearoylsphingosine |
